# A case study on chemistry classroom practices in the Rwandan secondary schools

**DOI:** 10.1016/j.heliyon.2021.e07352

**Published:** 2021-06-18

**Authors:** Edwin Byusa, Edwige Kampire, Adrian Rwekaza Mwesigye

**Affiliations:** a African Centre of Excellence for Innovative Teaching and Learning Mathematics and Science (ACEITLMS), University of Rwanda College of Education (URCE), Kayonza, P.O Box: 55 Rwamagana, Rwanda; b University of Rwanda College of Education (URCE), School of Education Kayonza, P.O Box: 55 Rwamagana, Rwanda; c Mbarara University of Science and Technology (MUST), Department of Educational Foundation and Psychology. Mbarara, Uganda

**Keywords:** Chemistry, Classroom practices, COPUS, Instruction, Rwandan schools

## Abstract

This study aims at assessing the classroom practices of Rwandan chemistry teachers in secondary schools, the second grade (S2), age range between 14 to 16 years old. The classroom observation conducted using the Classroom Observation Protocol for Undergraduate STEM (COPUS). In general, the best practice of group work or collaborative learning and students' engagement was observed. The analysis of the COPUS data reveals that active learning in chemistry classes is dominating, 54% against 42% of passive learning; found high, statistically significant over passive learning at *p* < .01 (tCritical = 1.89, df = 7, *p* = .003). The active students' practice is taking 82% of a 2-min time interval across 30 lessons observed, as one class period has 40 min to make 20 intervals of 2 min. Teachers are engaging their students in collaborative learning by assigning them various tasks in groups. These results established the current instructional practices in chemistry classes to draw conclusions and recommendations.

## Introduction

Quality education is a universal goal, and a lot is being done towards it worldwide. Classroom practice is among the different elements that will make it happen or not. Good classroom practice is part of a positive learning environment. Classroom practice is defined as what is happening within the classroom regarding actions to do with the teaching and learning process (Li and Oliveira, 2015). Students' performance can be improved through classroom practice when appropriate instructional strategies are used. A study conducted in Nigeria reveals that the cooperative instructional strategy improves learning outcomes in chemistry than the conventional teaching method (Aluko, 2008). It even reduces the students' anxiety in chemistry classes than chalk and talk strategy (Oludipe et al., 2010; Yusuf, 2014). However, we can still observe some instructional practices that are dominated by teacher-centred pedagogy. A study conducted in rural schools in Indonesia, in lower secondary grades, found that the teacher-centred approach was used with students limited in writing notes given by the teachers in science classes (Wahyudi and Treagust, 2004). Chemistry teachers are struggling to find an effective instructional practice that promotes improved learning outcomes in chemistry (Pawlak and Gross, 2019).

In general, classroom practices can be influenced by many factors at the teacher, individual level, or external factors (Drechsler, 2007). The selection of a good classroom practice promotes effective classroom management. Effective classroom management improves school attainment in science education (Pawlak and Gross, 2019). Students should be involved in all classroom activities as stakeholders to have improved interest in learning chemistry and improved school performance in chemistry (Agwuudu, 2017; Ejidike and Oyelana, 2015).

The study conducted in Rwanda informs that chemistry teachers prioritize group work-related activities (Byusa et al., 2020). Teaching chemistry and the development of other expected learning outcomes to do with values and attitudes that are associated with the lesson taught as teachers try to meet the expectation of the Competence-Based Curriculum (CBC) (REB, 2015b) needs classroom instructional practices that motivate learners the most to like the chemistry daily class activities.

Our study uses social constructivism as a learning theory developed by Lev Vygotsky (Vygotsky and Cole, 2018; Vygotsky, 1978). It is a sociological theory of knowledge that considers human development to be socially situated and the ability to be constructed through interaction with others (Boudry and Buekens, 2011; Palincsar, 1998; Powell and Kalina, 2009). Thus, learners are expected to learn well in groups or collaboratively. Our study is based on observing learners' learning through an active environment; thus, this theory fits in, revealing teaching and learning practices dominating passive and dynamic practices that engage students. Therefore, how the teacher interacts with students, how students interact with each other, and how the teacher engages students in useful activities may be well explained by the social constructivism theory.

In support of the group work-related activities, some chemistry teachers in Rwanda have started to engage learners more in the classroom activities using the approach of the physical embodiment of abstraction of the chemical phenomena; conceptual connections, and situated cognition/embodied cognition with a particular focus to the activity-based teaching techniques for the effective teaching of chemistry (Overmann and Malafouris, 2017; Perry and Medina, 2011). Collaboration among students improves conceptual understanding (Ndihokubwayo et al., 2020a). The collaboration of students is the building foundation of active learning. For instance, Fung et al. (2018), during analyses of the pre and post-diagnostic assessments, found that group work comprising effective strategies raised students' test scores and enhanced the joint construction of conceptual knowledge in science. This study provides insight into how far chemistry teachers have gone in less than five years, from teacher-centered to learner-centered pedagogy in grade 8 or secondary 2 (S2) classes. It will also serve as an outlook to policymakers to design policies and provide tools according to the needs of total engagement of learners in chemistry classes by making the classroom experience fully learner-centered. Therefore, the following is the research question that guided this study around chemistry teaching in the second grade two of secondary education: "What classroom practices dominate the Rwandan chemistry classroom?"

## Materials and methods

### Classroom Observation Protocol for Undergraduate STEM (COPUS) instrument and sampling procedures

This study uses classroom observation data collected in 10 secondary schools of Gasabo district, Rwanda. We selected these schools in March 2019 as guided by local authorities in charge of education during the meeting to have the district representation in the district's urban and rural areas. We collected the data from May 27th to July 8th, 2019, based on purposive sampling (Fraenkel et al., 2012). We focused on teachers teaching in chemistry in senior-2 (S2). All schools are day schools—we have preferred day schools over boarding schools to keep the same school characteristics. Boarding and day schools have some differences in infrastructure and structure, although they serve the same purpose. For instance, students at boarding spend the night at school. In contrast, students are limited in study concentration at day school as they spend day time at school and night at their homes (Ndihokubwayo et al., 2020b).

We used the COPUS to observe ten classes from 10 different teachers, one teacher per school, teaching 1610 students. In each of the ten schools, we observed three chemistry lessons. Thus, we observed each of 10 teachers thrice, making up 30 observed classes. The duration of the class period is 40 min in secondary schools in Rwanda. COPUS was developed by Smith et al. (2013) based on the Hora et al. (2013) study. This protocol helped us observe teachers' and learners' activities during the chemistry classes. COPUS was proven to be valid and reliable (Smith et al., 2013) and was used in the context of Rwandan secondary schools, taking into account its practical use. Even though COPUS was designed for undergraduate education, Akiha et al. (2018) and Ndihokubwayo et al. (2020b) demonstrated its relevance in secondary schools, which raised our confidence in its use in lower secondary schools.

This protocol comprises 25 codes related to teacher and student activities and three codes related to student engagement. Any observed code is rated in a 2-min time interval during the live classroom. Teacher activities codes are Lec-Lecturing; RtW-Realtime Writing; FUp-Follow-up questions; PQ-Posing non-clicker question, CQ-Posing Clicker question, AnQ-Answer questions, MG-Moving in the classroom and Guiding students; 1o1-One-on-one teacher support; D/V-teacher making demonstration by experimenting, simulation, etc., Adm-Administrating or giving feedback on tests; and W-Waiting during organizing materials of fixing tools such as a projector. The students' codes are L-Listening, AnQ-Answering teacher's questions, SQ-Asking question, WC-Whole-Class discussion, SP-Presentation of findings, In-Individual thinking, CG-Group work with Clickers, WG-Group working using worksheets, OG-Other Group, Prd-Prediction, T/Q-Test/Quiz, W-Waiting, and O-Other (Smith et al., 2013).

Before making observations, we underwent COPUS training, where the first author collaborated with one secondary school teacher with a strong background in chemistry and education. We discussed all the COPUS codes within 3 h. After three days, we gathered to code one chemistry lesson. We calculated interrater reliability and got a .94 coefficient. This factor is considered a very high agreement between two raters (Cohen, 1988; Landis and Koch, 1977; Smith et al., 2013). Concerning ethical clearance, the faculty members at the University of Rwanda College of Education (URCE) examined our research proposal, and the unit of research and innovation at the URCE issued us an ethical clearance—Ref:01/P-CE/567/EN/gi/2019—on March 18^th^, 2019. We submitted that clearance to the Gasabo district applying for data collection. We presented the approvals and permissions to the sampled schools. Before observing classes, we explained our study's purpose and scope to teachers and students and obtained verbal consent before the data collection.

### COPUS data analysis

**Figure 1:**
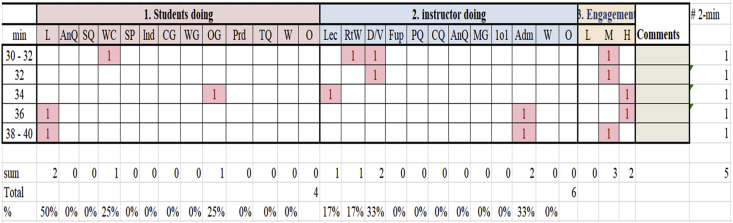
COPUS visualisation.

Figure 1 shows the data entry and analysis procedures. The marked "1" shows observed activity (COPUS code) during a specific 2-min time segment. As discussed, teacher and student codes, the student engagement (see column 4, Figure 1) is coded optionally to rate the extent of this engagement. For instance, the coder of this section did not find any 2-minute interval attributed to low engagement. He saw, however, teachers have three medium and two high engaged students in five 2-min time segments. According to COPUS developers (Smith et al., 2013), low engagement happens when a small fraction (10–20%) of students are engaged, medium engagement when a substantial fraction both engaged and not engaged, while high engagement is when a significant fraction of students (80%+) engaged in-class activity or listening to the teacher.

We followed the analysis procedure outlined by Ndihokubwayo et al. (2020). We used two methods to analyze the data, relative abundance (Lund et al., 2015; Smith et al., 2013) and relative frequency (Smith et al., 2014; Stains et al., 2018). To determine the relative abundance of each COPUS code, we added the total number of times each code was marked and divide it by the total number of codes, resulting in a percent of code. In this small section of our data (Figure 1), students coded listening (L) 2 out of 5 times, whole-class discussion (WC) once, and working in other groups (OG) once in five intervals or segments. Therefore, the total observed activities were 4. To find abundant activities, we computed the activity percentage. Thus, L was observed 50%, WC 25%, and OG 25%. Concurrently, the teacher was coded six times in 10 min (from 30 to 40 min interval) and was observed doing four activities. These activities were lecturing (Lec) observed in 2 out of 10 min or once in 5 segments, real-time writing (RtW) once, demonstration (D/V) twice, and administration (Adm) twice.

The relative frequency was computed to interpret data when multiple COPUS codes were marked simultaneously, which impacted the denominator of the calculation (Lewin et al., 2016; Lund et al., 2015). The "# 2-min" column contains the number of 2-minute time intervals coded. Thus, the coded 10 min from 30 to 40 min (see first column, Figure 1) has five segments. Therefore, the relative frequency was analyzed by taking the frequency of activity along a 2-min time interval. For instance, 40% of a 2-min time interval was spent on L, 20% on WC, while 20% was spent on OG. Similarly, 20% of activities were spent on lecturing (Lec), 20% on real-time writing (RtW), 40% on demonstration (D/V) twice, and 40% on administration (Adm). One can realize that this analysis does not add up to 100% like activity percentage (provided by relative abundance); the time interval percentage (provided by relative frequency) can go below or beyond 100%. The comments box is reserved for explaining difficult coding choices, flag key points for feedback for the teacher, identifying useful analogies, etc.

Since analyzing each code is difficult for forecasting classroom behavior such as student engagement, active learning, or lecture-based class, or passive learning, Smith et al. (2014) have proposed to collapse the codes into small groups. These groups are eight in total, four for teachers, and four for students (see Table 1).

**Table 1:** Collapsed codes, as suggested by Smith et al. (2014).

Collapsed codes	Individual codes
**Teacher activities**	
Presenting (P)	Lec: Lecturing or presenting information
	RtW: Real-time writing
	D/V: Showing or conducting a demo, experiment, or simulation
Guiding (G)	FlUp: Follow-up/feedback on clicker question or activity
	PQ: Posing a non-clicker question to students (nonrhetorical)
	CQ: Asking clicker question (entire time, not just when first asked)
	AnQ: Listening to and answering student questions to the entire class
	MG: Moving through class guiding ongoing student work
	1o1: One-on-one extended discussion with individual students
Administration (A)	Adm: Administration (assign homework, return tests, etc.)
Other (OI)	W: Waiting (instructor late, working on fixing technical problems)
	O: Other
**Students' activities**	
Receiving (R)	L: Listening to the instructor
Talking to class (STC)	AnQ: Student answering question posed by the instructor
	SQ: Student asks the question
	WC: Students engaged in a whole-class discussion
	SP: Students presenting to the entire class
Working (SW)	Ind: Individual thinking/problem solving
	CG: Discussing clicker question in groups of students
	WG: Working in groups on worksheet activity
	OG: Other assigned group activity
	Prd: Making a prediction about a demo or experiment
	TQ: Test or quiz
Other (OS)	W: Waiting (instructor late, working on fixing technical problems)
	O: Other

We present our data in collapsed codes form, and our grouping is based on social constructivism theory (Vygotsky and Cole, 2018) that emphasizes group work. To do this, we revisited the collapsed students' codes by splitting the "talking to class" group and merge its parts into the "working" group. Many authors have classified group work (Akiha et al., 2018; Fung et al., 2018) and questioning (Fang, 2020; Lewin et al., 2016; Stains et al., 2018) as active learning. Thus, our students collapsed groups became active learning (see Table 1). However, the teacher's codes stayed untouched. We employed inferential statistics to support descriptive data. We analyzed the variances (Anova: Single-factor) between groups (Receiving, Talking to class, and Working) and within groups (see Table 1). We computed t-Test (Two-Sample Assuming Equal Variances) to check statistical significance between 'Presenting and Guiding' and between 'Active and Passive learning.'

## Results and discussion

The results of the Classroom teaching and learning practices documented with the COPUS tool show that teachers' time in the chemistry classroom is dominated by presenting chemistry-related knowledge, skills, and associated values and attitudes as stipulated in Table 2. Teachers spend much of their time lecturing, demonstrating, and writing on the board. This dominance is shown by 73% of a 2-min time segment. Concurrently, students spend 40% of a 2-min time segment receiving and listening to the instructor during teacher presentations. Teachers spend 61.7% of a 2-min time segment guiding students, who spend their time working as shown by 54% of a 2-min time segment while talking is 52%. Not only our study but different studies have shown similar results. For instance, Stains et al. (2018) have found didactic over interactive instructional style. Socratic and lecture methods dominated other classroom activities; for instance, they found Socratic and lecture methods in 184 class periods, while peer instruction and collaborative learning only appeared in 85 class periods.

**Table 2:** Teacher and student time spent on collapsed codes (relative frequency). Activity as a Percentage of Time Intervals.

	Activities	Activity %	Standard deviation
Collapsed Codes (Students)	Receiving	40%	0.00
	Talking to class	52%	0.06
	Working	54%	0.10
	Other (Students)	5.4%	0.00
Collapsed Codes (Teacher)	Presenting	73%	0.05
	Guiding	61.7%	0.10
	Admin	5.4%	0.00
	Other (Teacher)	14.4%	0.05

**Figure 2:**
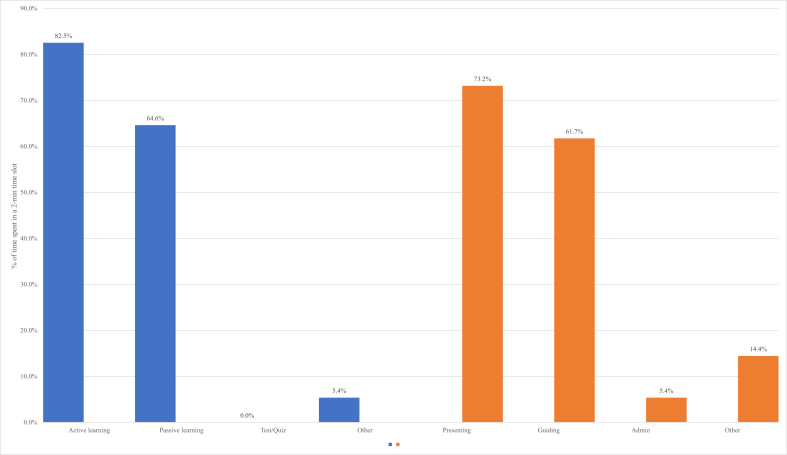
Students and teacher time spent on classroom practices (relative frequency). Activity as a Percentage of Time Intervals.

We merged receiving and individual thinking (Ind) to know the classroom practices, and we found passive learning consumes 64% of a 2-min time interval (see Figure 2). However, active learning dominates passive learning as it consumes 82% of a 2-min interval. There was a statistically significant difference between Receiving/Talking to class and Working on the side of students at p < .05 (F = 5.17, df = [2,7], *p* = .04). Guiding was statistically significant over Presenting on the teacher's side at *p* < .05 (tCritical = 1.94, df = 6, *p* = .04). Therefore, active learning was found high, statistically significant over passive learning at *p* < .01 (tCritical = 1.89, df = 7, *p* = .003). The active learning was computed on merging talking to class and working activities excluding "Ind" and "T/Q" codes. Active learning (82%) is higher than teacher presenting (73.2%). It might be caused by the training provided to teachers from the implementation of a new competency-based curriculum (Ndihokubwayo et al., 2019; Ndihokubwayo and Murasira, 2019; REB, 2015). This training was provided to teachers in Rwanda in phases. So far, three phases have been completed with continuous training to equip teachers with the required methodological skills to be better able to teach and to build competencies in their students.

**Figure 3:**
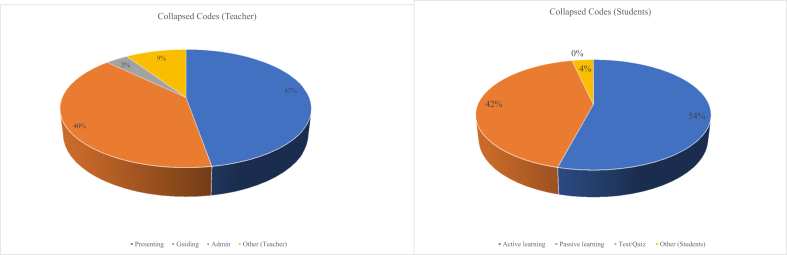
Results of the classroom observation about both teachers' and students' activities percentage. Teachers activities: Presenting = Lec, RtW, D/V, Guiding = FUp, PQ, CQ, AnQ, MG, 1o1; Admin = Adm; Other = W, O.

Apart from Table 2 and Figure 2, we triangulated our analysis. We can visualize each activity happening in a classroom using relative abundance (see Table 3 and Figure 3). Table 3 presents the collapsed codes (Smith et al., 2014) as activity percent, cumulative percent, and standard deviation. Each activity was computed by taking the sum of related codes. Each teacher's or student's activities add up to one hundred. For instance, the receiving, talking to the class, working, and others make students' collapsed codes, and all of them add up to 100%. This analysis allowed us to measure the portion of each activity in comparison to others. The students working are emerging activity as it contributes to 36% of their related activities. Thus, the teacher assigns enough activities to students and allows them to work in groups. Teacher presenting emanate from other activities as it contributes to 47% of other related activities. This behavior was caused by lecturing or talking, writing on the board, and demonstrating a phenomenon or doing an experiment. Although this demonstration should shift from the teacher's doing to the learner, it shows how teachers are motivated to make their teaching clear and make their students understand by varying talking and demonstrating.

**Table 3:** Teacher and student activity percentage (relative abundance). All teachers' activities or students' activities add up to 100% cumulatively.

	Activities	Activity %	Standard deviation
Collapsed Codes (Students)	Receiving	26%	0.00
	Talking to class	34%	0.04
	Working	36%	0.06
	Other (Students)	4%	0.00
Collapsed Codes (Teacher)	Presenting	47%	0.03
	Guiding	40%	0.06
	Admin	4%	0.00
	Other (Teacher)	9%	0.03

During their study of active learning across the level of education (Akiha et al., 2018), they argued for the role of laboratory activities in middle schools. The authors found that while laboratory activities are scheduled in universities, they are within the course structure in middle schools. This is encompassed by the same period where students are working (36%) and teacher guiding (40%). It shows that teachers allocate the same effort in assigning tasks to students and guiding them to fulfill the given task. Our study correlates with one done using the Reformed Teaching Observation Protocol (RTOP). The authors found that chemistry teachers developed their students through inquiry methods (Rushton et al., 2011). This fact shows the importance of questioning as an effective learning method. Other studies, apart from chemistry, showed good progress in implementing the competence-based curriculum that adapts to active learning in Rwandan classrooms. For instance, Ndihokubwayo et al. (2020a) found that the physics classroom is reformed at 53%. Collaborative learning is integrated at 61% for students at day schools, which is used more than their counterparts in boarding schools (Ndihokubwayo et al., 2020b).

The classroom observation in selected schools, using the COPUS observation protocol, reveals that teachers are progressing well toward the total engagement of learners in chemistry classrooms. For instance, active learning stands at 54% over 42% of passive learning of all the activities under students' collapsed codes in chemistry classes (Figure 3). There was a statistically significant difference between Receiving, Talking to class, and Working at significance level *p* < .05 (*F* = 5.17, df = [2,7], *p* = .04) on the side of students. Guiding was statistically significant over Presenting at p < .05 (tCritical = 1.94, df = 6, p = .04) on the teacher's side. Therefore, active learning was found high, statistically significant over passive learning at *p* < .01 (tCritical = 1.89, df = 7, *p* = .003). Our study concurs with one of Akiha et al. (2018), who surveyed middle school teachers on the role of active learning. Teachers said that "actively encouraged in the middle and high school level as part of our understanding of best practices in pedagogy (Akiha et al., 2018, p. 13). "However, our study has shown a different perspective among related studies, which studied active learning and used COPUS protocol. We found that both teacher and student activities are correlated in the same range, and no time is spent more on activity than another. For instance, Smith et al. (2014) found that some classes experience up to 94% of lectures during the 2-min time interval. Akiha et al. (2018) experienced some classes with students receiving from 0 up to 100% of a 2-min time interval.

By looking at learners and teacher's activities' percentage and how they spend time on those activities, it is clear that the majority of activities of learners during the chemistry classrooms in S2 were dominated by engaging activities, which imply the collaboration of learners and the excellent interaction with the teacher during the teaching and learning activities. From the social constructivism theory (Vygotsky, 1978), interaction impacts people's active learning. Therefore, chemistry teachers in Rwanda should enhance this together with the incorporation of an active teaching approach that promotes the students' curiosity in doing chemistry It was found that curiosity in subject matter contributes a lot to learn that subject (Gurning and Siregar, 2017; Higgins and Moeed, 2017; Luce and Hsi, 2015; Savlin, 1998). Otherwise, it can hinder the importance of collaborative learning and active learning.

The students' compromise between themselves and supported by teachers should be put into place (Savasci and Berlin, 2012) as group work and sharing concerns from the classroom are key to improve teaching and learning in the classroom. Thus, this will be due to the directed content discussion and learning objectives, as well as reflection on teacher and learner roles in the classroom (Van Duzor, 2012). Otherwise, this would discontinue the will of the current curriculum implemented in Rwanda from 2016. The curriculum is competency-based and emphasizes getting and using skills of what has been learned (Ndihokubwayo and Murasira, 2019; REB, 2015). Therefore, it aims at shifting from individual-focused and passive learning to collaborative and active learning.

## Conclusions and limitation of the study

Our case study shows that teachers in Rwanda use active learning by involving learners in work and guiding them on assigned work. Active learning was documented at 54% of other activities alongside passive learning at 42%, Test/Quiz at 4%. Passive learning was considered as listening and individual work. In contrast, active learning comprised activities related to collaborative learning such as group work and activities related to questions such as students asking and answering questions and activities related to reporting the findings, such as communicating and sharing ideas with the whole class.

Therefore, chemistry teaching in S2 can take full advantage of group work-related activities, the use of the physical embodiment, and situated cognition/embodied cognition through activity-based techniques to maximize the students' interest in studying chemistry. The students' collaboration should be empowered and curious to discover the world during the teaching and learning process.

The data collected for this manuscript is solely on observation of how time is allocated in the classroom on different teaching and learning modalities and not comparing teaching and learning methods with learning assessments. To remediate this and avoid the overreaching conclusion, we have limited our paper to only time allocation on different classroom practices. Our active versus passive learning only combines some COPUS practices; we compare the combined codes related to active learning to induce passive learning. But, we do not compare active learning with learning outcomes or students' performance. Our study was limited to only ten schools. Therefore, future research should focus on more schools. The analysis should be done by observing many classes and seeking more and various views from both teachers, students, and school managers.

For more improvements in academic performance, we recommend including more activities or using the strategy that will promote students' curiosity when doing chemistry or any other science. In this regard, the classroom practices and their link to students' academic performance should be of interest for future studies.

## Declarations

### Author contribution statement

Edwin Byusa: Conceived and designed the experiments; Performed the experiments; Analyzed and interpreted the data; Wrote the paper.

Edwige Kampire, Adrian Rwekaza Mwesigye: Conceived and designed the experiments.

### Funding statement

This work was supported by The African Center of Excellence for Innovative Teaching and Learning Mathematics and Science (ACEITLMS)

### Data availability statement

Data associated with this study has been deposited at Mendeley under https://data.mendeley.com/datasets/j6h4xsj34k/1.

### Declaration of interests statement

The authors declare no conflict of interest.

### Additional information

No additional information is available for this paper.
